# Advancing Early Dementia Diagnosis: Insights From Community-Based Studies in Korean American Older Adults

**DOI:** 10.1093/geroni/igaf122.361

**Published:** 2025-12-31

**Authors:** Hae-Ra Han

## Abstract

With the rapid growth of the aging population, disparities in dementia diagnosis and care persist, particularly among individuals with limited English proficiency (LEP), such as Korean Americans. Early detection improves quality of life, reduces healthcare costs, and enhances the benefits of emerging disease-modifying therapies. However, limited dementia literacy and language barriers often delay diagnosis and treatment. This symposium presents insights from two community-based studies on Korean American older adults—MASK-MD (Memory and Aging Study of Koreans in Maryland), an ethnic church-based cognitive assessment study, and PLAN (Preparing for health aging through dementia Literacy And Navigation), a multi-site, community-based randomized controlled trial. Discussions will cover: 1) Findings from MASK-MD which screened over 1,000 older Korean Americans in ethnic churches in Maryland, along with key insights from the PLAN trial, such as 2) unique barriers and facilitators related to recruiting and retaining community-dwelling Korean American older adults with undiagnosed dementia and their caregivers, 3) the impact of digital literacy on social isolation in the sample, and 4) findings from PLAN’s outcomes analyses, focusing on its effectiveness in promoting linkage to medical services for formal dementia evaluation and care planning. This symposium aims to strengthen the evidence base on dementia prevalence and strategies for promoting early diagnosis and care planning among LEP older adults in community settings. These findings are critical for addressing health inequities through behavioral and social science research.

